# Fiber-Optic Probe Array for Measuring Spatial Distributions of Air Volume Fractions in Bubbly Flows

**DOI:** 10.3390/s23031222

**Published:** 2023-01-20

**Authors:** Tsung-Mo Tien, Ching-Jer Huang, Chien-Hsun Lee, Kuan-Wen Liu

**Affiliations:** 1 Coastal Water and Environment Center, National Kaohsiung University of Science and Technology, Kaohsiung 81157, Taiwan; 2 Department of Hydraulic and Ocean Engineering, National Cheng Kung University, Tainan 70101, Taiwan; 3 Coastal Ocean Monitoring Center, National Cheng Kung University, Tainan 70101, Taiwan; 4 River and Coast Division, Water Resources Agency, Ministry of Economic Affairs, Taichung 408281, Taiwan

**Keywords:** fiber-optic probe array, local air volume fraction, bubbly flow, breaking waves

## Abstract

In this study, we developed a fiber-optic sensing system with an eight-probe array for measuring the spatial distributions of air volume (void) fractions in bubbly flows. Initially, we performed calibration experiments in a cylindrical tank by using a fiber-optic sensing system with a single probe to determine the relationship between the time fraction ratio of bubble signals and void fractions. A high correlation coefficient was obtained between the time fraction ratio and the void fraction, suggesting that the proposed fiber-optic sensing system can measure local void fractions of up to 18%. Subsequently, we used the proposed fiber-optic sensing system with the eight-probe array to measure the spatial distribution of air volume fractions in a bubbly flow caused by breaking waves near a submerged breakwater. The effects of different variables, including the incident wave height, period, and width of the breakwater, on the spatial distribution of the void fraction on the lee side of the breakwater were systematically studied. The results demonstrated that the proposed fiber-optic sensing system can be used to determine the spatial distribution of air volume fractions in bubbly flows.

## 1. Introduction

Bubbly flows are defined as liquids that contain a certain quantity of gas bubbles. Bubble size, bubble velocity, bubble population, bubble size distribution, and local air volume fraction are critical physical properties associated with bubbly flows. Air volume fraction, also called void fraction, is defined as the ratio of the air volume to the total volume of air and liquid in a bubbly liquid sample.

Acoustic and optical techniques are traditionally applied to determine the aforementioned physical properties associated with bubbly flows [1,2,3,4,5,6]. Medwin [1,2] has proposed the resonator method for estimating the bubble population by measuring the backscatter and attenuation of sound propagating through a bubbly liquid. Deane [3] used photography to measure high void fractions and detect large bubbles in the surf zone. Vagle and Farmer [4] compared four methods for measuring bubble size and void fraction and demonstrated that single-frequency and multi-frequency backscatter sonars used in the resonator method cannot resolve areas with high bubble concentrations, especially the surf zone. Leighton et al. [5] proposed a technique for determining bubble size and numbers from underwater noise. Deane and Stokes [6] provided a quantitative description of bubble formation mechanisms by capturing high-speed video images and detecting underwater noise produced by a breaking wave. They concluded that bubbles larger than approximately 1 mm (i.e., those beyond the Hinze scale) are subject to fragmentation caused by turbulent shear flows and exhibit a −10/3 power-law scaling with respect to the radius. Furthermore, bubbles smaller than the Hinze scale are stabilized by surface tension and exhibit a −3/2 power-law scaling with respect to the radius.

Recently, fiber-optic probes have been extensively used to study the bubble velocity, bubble size, and local void fraction in bubbly flows [7,8,9,10,11,12,13,14,15]. Local air volume fractions in bubbly flows can be estimated by detecting phase variations in the probe tip. Fiber-optic probes are characterized by their fine structure, which minimizes the disturbances of a device to a physical field.

The bubble signature can be identified by examining the phase variations detected by the applied fiber-optic probes. However, the features of the bubble signature heavily depend on the probe–bubble interaction. Cartellier [7] identified four characteristics associated with the positions of bubbles relative to the probe tip. Barrau et al. [16] refined the classification of probe–bubble interactions for fiber-optic probes to include the blinding, crawling, and drifting effects. These effects may influence local void fraction estimates. Chang et al. [17] demonstrated the feasibility of using a fiber-optic reflectometer to determine the velocity and fraction ratio of solid particles and gas bubbles or liquid droplets in liquid or gas flows. Juliá et al. [12] investigated the accuracy of optical probes in measuring void fractions in bubbly flows. They devised a criterion for determining the accuracy of the probes and the effects of probe–bubble interactions on residence time estimation by comparing optical probe signals with charge-coupled device images. Rojas and Loewen [13] determined the accuracy of single-tip fiber-optic probes in simultaneously measuring void fraction and bubble size distribution under breaking waves.

Blenkinsopp and Chaplin [18] measured the time-varying void fraction distribution in laboratory breaking waves by using a single fiber-optic probe. Similar to this experimental setup, Blenkinsopp and Chaplin [14] used a pair of fiber-optic probes to measure the size of bubbles entrained by breaking waves and revealed the spatial and temporal evolution of bubble sizes within a breaker. The spatial variation of the bubble sizes was obtained by moving the probe at 20 mm vertical intervals along four vertical transects (longitudinal direction of the wave flume) in the bubbly flow region. Vejražka et al. [15] investigated the effect of the intrusive nature of a single fiber-optic probe on its accuracy in measuring bubbly flow characteristics and demonstrated that the void fraction in a flow can be correlated with a modified Weber number. Furthermore, this correlation is helpful for sensor selection and uncertainty estimation. Besagni et al. [19] and Na et al. [20] have combined fiber-optic sensing techniques with image analysis methods to determine the bubble size distributions and void fractions in bubbly flows.

In addition to single-tip fiber-optical probes, dual-tip phase detection probes have been used to estimate the interfacial velocities and void fractions in bubbly flows and highly aerated flows [16,21,22,23,24,25,26]. In a cross-correlation analysis, signals from the two probe tips were simultaneously sampled, and the results indicated that their interfacial time-averaged velocity was reliable. Alternatively, the average bubble velocity can be determined by dividing the distance between the tips of the bi-probe (usually 0.6–3 mm) by the time taken by the interface to pass from one probe to the other.

Do and Chang [27] applied a single-probe fiber-optic reflectometer to identify water, air bubbles, and oil droplets and to determine the velocity and size of the bubbles and droplets in an oil–gas–water three-phase flow by processing the measured signals. Wang et al. [28] investigated the characteristics of an air–water slug flow in a vertical tube by employing single and double optical probes, high-speed photography, and random signal analysis. Extensive data on the local void fraction, bubble velocity, and length of Taylor bubbles and liquid slugs were obtained. Lefebvre et al. [29] proposed a new, optimized Doppler optical probe for detecting air phase and measuring bubble velocity and size in complex bubbly flows.

The spatial distribution of the air volume fraction in a bubbly flow is crucial for determining the attenuation of sound propagating through the bubbly flow [30,31]. However, limited studies have been conducted on the determination of the spatial distribution of the air volume fraction in a bubbly flow. Conventional acoustic and optical techniques can be used to determine spatially averaged void fractions from underwater noises and video images, respectively. An array of fiber-optic probes may provide the spatial distribution of void fractions; however, limited studies have been conducted on such probes.

The present study developed a fiber-optic sensing system comprising a movable eight-probe array for determining the spatial distribution of air volume fractions in bubbly flows. By moving the vertical probe array horizontally, the spatial distribution of air volume fractions in the desired bubbly flow regions can be determined.

The remainder of this paper is organized as follows. Section 2 presents the fiber-optic sensing technique for measuring the air volume fraction in bubbly flows. Calibration experiments for determining the relationship between the time fraction ratio of bubble signals and the local void fraction ratio in a bubbly flow are presented in Section 3. Section 4 proposes a fiber-optic sensing system with an eight-probe array for measuring the spatial distribution of air volume fractions in a bubbly flow at the rear of a submerged breakwater. Section 5 provides the results and discussion regarding the characteristics of the measured bubbly flow. Finally, the conclusions of this study are presented in Section 6.

## 2. Fiber-Optic Sensing Technique for Measuring Air Volume Fractions in Bubbly Flows

In the conventional two-phase flow model [32], local void fractions are defined using the gas-phase density function $X_{G}(\underline{x},t)$. The parameter $X_{G}$ is equal to 1 whenever the measurement position, $\underline{x}$, is located in the air phase at moment t; otherwise, $X_{G}$ is equal to 0. The local time fraction ratio of the gas phase $\alpha (\underline{x})$ in a given time interval (*t′*, *t′* + *T*) at $\underline{x}$ is expressed as follows:

(1) $$\alpha (\underline{x})=\underset{T\to \infty }{\lim}\;[\int_{t^{\prime }}^{t^{\prime }+T}X_{G}(\underline{x},t)dt/T]$$

where T is the measurement time. If the gas residence time, $T_{Gi}$, is introduced into the aforementioned equation, $\alpha (\underline{x})$ can be expressed as follows:

(2) $$\alpha (\underline{x})=\underset{T\to \infty }{\lim}\;[\sum_{i}T_{Gi}/T]$$

$T_{Gi}$ represents the duration from the start of the ascending slope to the start of the descending slope of the bubble signature when the probe tip pierces a bubble. The subscript *i* denotes the number of bubbles that are pierced by the probe tip. After the time fraction ratio of the gas phase is obtained, the air volume fraction can be determined from an experimentally derived calibration curve or the relationship between these two parameters.

**Figure 1:**
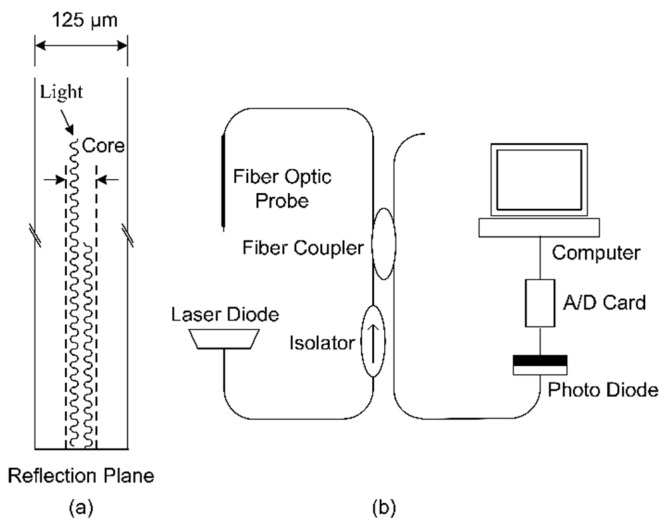
Schematic of the proposed fiber-optic sensing system for measuring local air volume fractions in bubbly flows: (**a**) light reflection at the probe tip and (**b**) experimental setup.

Figure 1 presents a schematic of the proposed fiber-optic sensing system for measuring the air volume fraction in bubbly flows. Figure 1a illustrates the reflection of optical signals at the probe tip, and Figure 1b displays the experimental setup, which consisted of the proposed system with a laser diode, an isolator, a 2×2 fiber coupler, a fiber-optic probe, and a photo diode. In this system, a single-mode fiber-optic cable was used and a fiber-optic probe was immersed in the bubbly flow to detect the phase variation at the probe tip. The laser diode emits a continuous optical signal with a wavelength of 1550 nm into a single-mode optical fiber. The diameters of the fiber core and cladding are approximately 8 and 125 μm, respectively.

The emitted optical signal is divided into two beams by the 2×2 coupler with a nominal coupling ratio of 0.5. The fiber-optic probe is placed into the bubbly flow for phase variation detection. The strength of the reflected optical signal depends on the phase of flow past the probe tip. The reflected signal returns through the original fiber and coupler and is directed into the photo diode, which converts the obtained optical signals into electrical signals. The electrical signals are then obtained using a data acquisition board and stored on a personal computer for further data processing. According to the Fresnel reflection equation, the power of the reflected optical signal at the probe tip can be expressed as follows [33]:

(3) $$p=p_{o}k^{2}{\left(\frac{n-n_{f}}{n+n_{f}}\right)}^{2}$$

where $p_{o}$ is the power of light emitted from the laser diode, k is the coupling ratio of the fiber coupler (k=0.5), n is the refractive index of the tested fluid, and $n_{f}$ is the refractive index of the fiber ($n_{f}\approx 1.44$). In bubbly flows, the refractive indices of air and water are approximately 1.0 and 1.33, respectively.

## 3. Calibration Experiments

**Figure 2:**
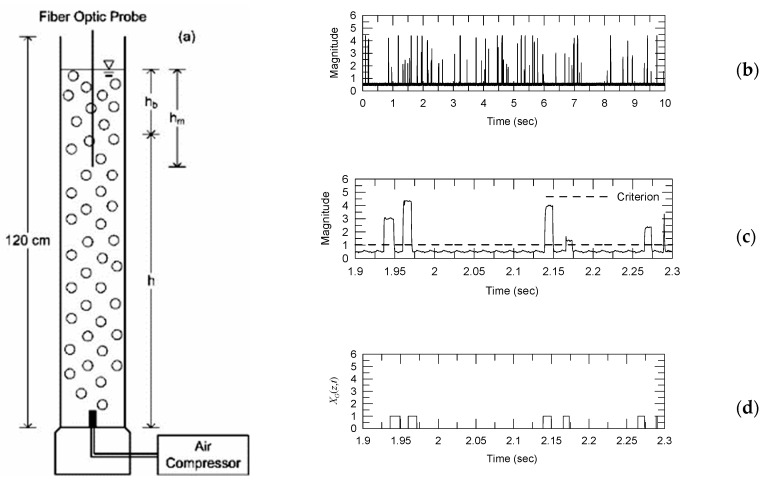
Calibration experiments for determining the relationship between the air volume fraction and the time fraction ratio of bubble signals: (**a**) experimental setup, (**b**) device output when $h_{b}=$ 5.60 cm and h= 83.25 cm, (**c**) signal in the time span from 1.9 to 2.3 s, and (**d**) gas-phase density function, $X_{G}(z,t)$, from 1.9 to 2.3 s at the measurement position. The magnitude of the optical signals is presented as strength in volts. The dashed line in (**c**) indicates the criterion for distinguishing the gas phase.

A single fiber-optic probe can be inserted into the bubbly flow to detect phase variation in the flow and determine the time fraction ratio of the bubble signals. However, to elucidate the relationship between the air volume fraction and the time fraction ratio of the bubble signals, calibration experiments were required. Figure 2a displays the vertical pipe used in the calibration experiments conducted in this study. This pipe had an inner diameter of 7.4 cm and a height of 120 cm. An air compressor with a valve for controlling the air-flow rate was used to pump air into the water in the vertical pipe.

Compressed air flowed through a porous medium located at the bottom of the vertical pipe, resulting in the formation of a bubble plume. In Figure 2a, h denotes the distance from the bottom of the vertical pipe to the free surface before the air inflow and $h_{b}$ represents the increased height caused by the inflow of air. As mentioned, the fiber-optic probe was placed into the bubbly flow to measure phase variations. The probe tip was positioned at a distance of 75 cm from the pipe bottom. The parameter $h_{m}$ represents the distance from the probe tip to the free surface. Each measurement in the bubbly flow was performed for 200 s, and the sampling rate was 100 kHz. The global void fraction in the bubbly flow in the vertical pipe was calculated using the following equation:

(4) $$\beta_{g}=\frac{h_{b}}{h+h_{b}}$$

Due to the compressibility of the air in the vertical pipe, the global void fraction differed from the local void fraction at the probe tip. The ideal gas law, in which the pressure acting on air bubbles is assumed to be hydrostatic pressure, was employed to convert the global void fraction into the local void fraction. The conversion procedure is described in the following text.

The bubbly water in the vertical pipe was divided into n equal vertical sections. Each cylindrical pipe section had a height of $(h+h_{b})/n$. $V_{i}$ and $V_{ia}$ represent the bubbly water volume and the air volume in the *i*-th section, respectively, and $P_{i}$ denotes the hydrostatic pressure acting on this section. Similarly, the corresponding variables for the fiber-optic probe section are denoted as $V_{m}$, $V_{ma}$, and $P_{m}$, respectively. The hydrostatic pressure acting on each section could be easily determined. Moreover, assuming the bubbles to be uniformly distributed within the bubbly liquid and the water temperature throughout the pipe to be constant, we can express the ideal gas law for an isentropic process as follows:

(5) $$PV^{\gamma }=\mathrm{constant}$$

where γ denotes the ratio of the specific heat at constant pressure ($c_{p}$) to the specific heat at constant volume ($c_{v}$). For air, γ=1.4. Based on Equation (5), we can obtain the following expression:

(6) $$P_{i}V_{ia}^{\gamma }=P_{m}V_{ma}^{\gamma }$$

or

(7) $$V_{ia}={\left(P_{m}/P_{i}\right)}^{-\gamma }\cdot V_{ma}$$

The total air volume in the pipe is denoted as $\sum_{i=1}^{n}V_{ia}$, which can be expressed as follows:

(8) $$\sum_{i=1}^{n}V_{ia}=\sum_{i=1}^{n}\left[{\left(P_{m}/P_{i}\right)}^{-\gamma }\cdot V_{ma}\right]=h_{b}\cdot A_{c}$$

where $A_{c}$ represents the cross-sectional area of the pipe. After determining $V_{ma}$ from Equation (8), we can derive the local air volume (void) fraction as follows:

(9) $$\beta_{m}=\frac{V_{ma}}{V_{m}}$$

The gas residence time, $T_{Gi}$, is required to determine the local time fraction ratio, $\alpha (\underline{x})$, through Equation (2). Figure 2b illustrates typical time-series data collected by the probe during the sampling time T=10 s when h=83.25 cm and $h_{b}=5.60\;\mathrm{cm}$. The optical signals in this figure represent the strength (in volts) of the reflected signal. The gas-phase density function, $X_{G}(\underline{x},t)$, was determined using a signal-processing method similar to that of Barrau et al. [16]. The criterion adopted for distinguishing the air phase was based on the low-level criterion proposed by Juliá et al. [12] and is depicted in Figure 2c. Numerous studies have applied this criterion to identify the air phase in a multi-phase flow [15,34,35,36]. To demonstrate the behavior of the bubble signals, the time-series data in Figure 2b from 1.9 to 2.3 s are detailed in Figure 2c. Figure 2d illustrates the results for $X_{G}(\underline{x},t)$, corresponding to those presented in Figure 2c. In Figure 2d, $X_{G}(\underline{x},t)$ is denoted as $X_{G}(z,t)$ to indicate that the measurement position, $\underline{x}$, is in the vertical (z) direction. The gas residence time, $T_{Gi}$, was calculated using $X_{G}(\underline{x},t)$. After the local $T_{Gi}$ value was obtained, the local time fraction ratio of gas phase, α, was determined using Equation (2).

**Figure 3:**
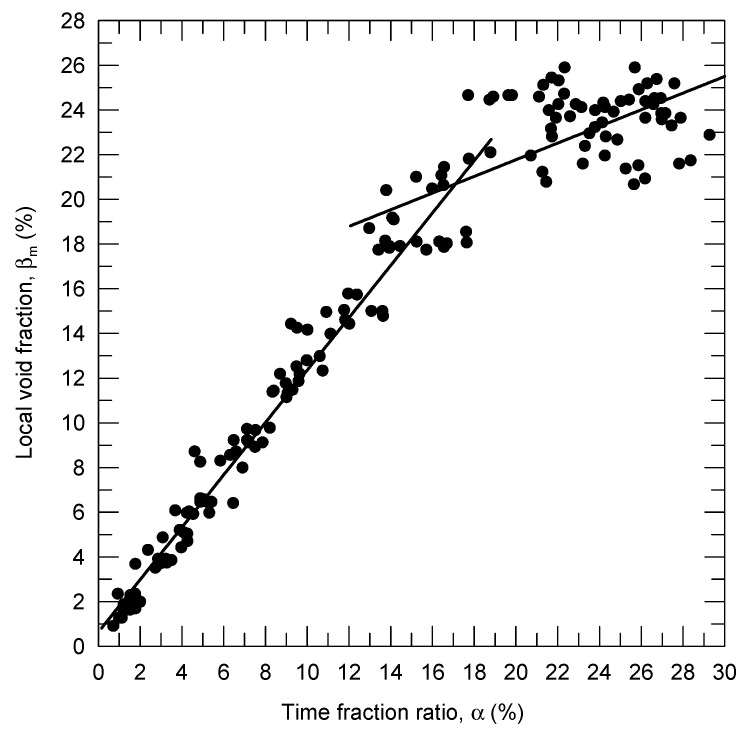
Relationship between $\beta_{m}$ and α for bubble signals.

Figure 3 presents the experimental data obtained for the local void fraction, $\beta_{m}$, with respect to α at various h and $h_{b}$ values. Based on the linear regression, the relationship between $\beta_{m}$ and α when α<17% was derived to be:

(10) $$\beta_{m}=0.0064+1.17\cdot \alpha$$

When α>17%, the following equation was obtained:

(11) $$\beta_{m}=0.2053+0.37\cdot (\alpha -0.17)$$

When α<17% ($\beta_{m}<20\%$), the correlation coefficient between α and $\beta_{m}$ was 0.95. By contrast, when α>17% ($\beta_{m}>20\%$), the correlation coefficient between α and $\beta_{m}$ was only 0.48 because the data were widely scattered around the linear regression line.

The possible reason for the low correlation coefficient between α and $\beta_{m}$ at a high air volume fraction is described as follows: To provide a high air-flow rate, the air compressor had to continually compress air to compensate for the decreasing air pressure caused by the release of air into the pipe. This process might have led to an unstable airflow output and induced a convection of the bubbly flow in the pipe. This flow convection in turn caused the non-homogeneity of the bubble distribution, thus resulting in the low correlation coefficient between α and $\beta_{m}$. Furthermore, the sampling time is also crucial for determining the rating curve between α and $\beta_{m}$. As presented in Table 1, when the sampling time increased from 1 to 3, 5, and 10 s, for α<17%, the linear regression approached the relationship presented in Equation (10) with a correlation coefficient of 0.95. According to Figure 3, when $\beta_{m}<18\%$, Equation (10) could be used to determine $\beta_{m}$ from α.

## 4. Setup of the Fiber-Optic Sensing System for Measuring Void Fraction Distribution

**Figure 4:**
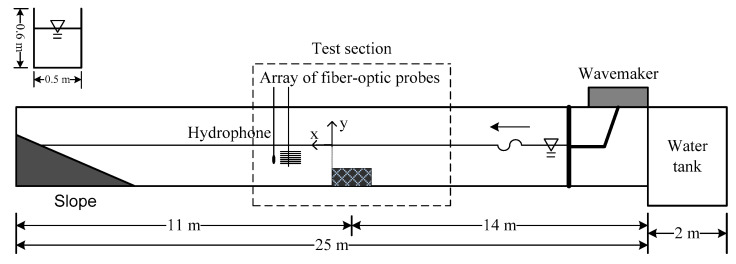
Experimental setup for measuring the spatial distribution of the air volume fraction in the bubbly flow caused by breaking waves near a submerged breakwater. The breakwater model was composed of Plexiglas and was placed in the middle of the wave flume.

After the relationship between the local void fraction and time fraction ratio was determined, a fiber-optic sensing system with an array of eight probes was proposed to determine the distribution of the air volume fraction in bubbly flows. The capability of the proposed system was demonstrated by using it to measure the spatial distribution of the air volume fraction in a bubbly flow near a submerged breakwater. This bubbly flow was induced by the propagation of water waves over a submerged breakwater under the condition of wave breaking. In general, when waves break, air is entrained into the water, which results in the formation of a bubble plume or bubbly flow. Figure 4 presents a schematic of the experimental setup for determining the spatial distribution of the air volume fraction near the submerged breakwater. Waves are produced in the wave flume by using a piston-type wavemaker, and these waves propagated from right to left. A two-dimensional breakwater model composed of Plexiglas was placed in the middle of the flume, and a sloping bottom was deployed at the end of the flume for reducing the reflected waves.

On the rear side of the breakwater, we deployed an array of eight fiber-optic probes to simultaneously measure the local air volume fractions at eight depths. The probe had a diameter of 245±10 μm, and the gap between two neighboring probes was 1 cm. The eight probes were mounted on a vertical arm to form a fiber-optic array. The vertical arm was suspended from a rod that was installed across the sidewalls of the wave flume. This array was positioned in the middle of the flume, and it could be moved horizontally or vertically to determine the air volume fraction distribution in a given region. Since most breaking waves occur on the rear side of a breakwater, we determined the air volume fraction in this region only.

**Figure 5:**
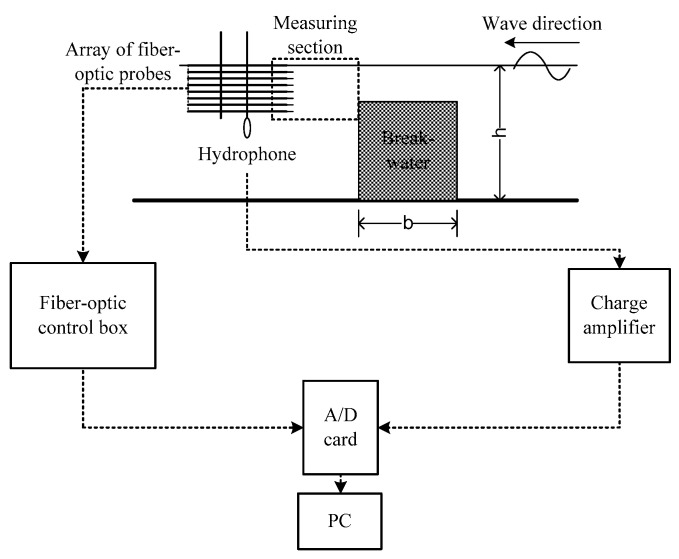
Measurements of the air volume fraction in the bubbly flow near a submerged breakwater by using the proposed fiber-optic sensing technique. b: breakwater length.

**Figure 6:**
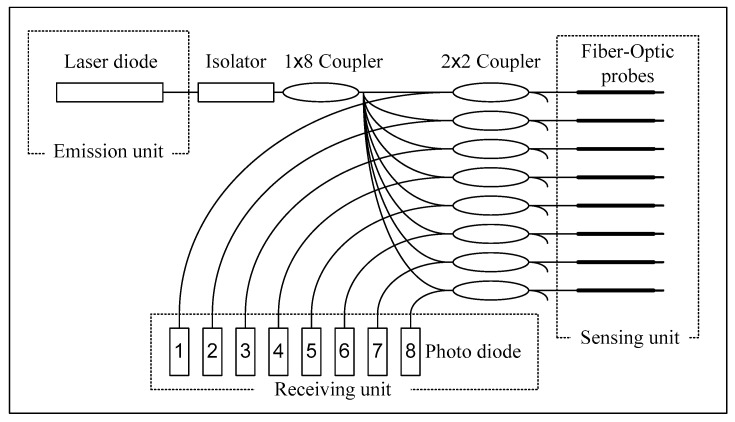
Emission, sensing, and receiving units in the fiber-optic control box.

A hydrophone was also installed near the fiber-optic probe array to provide additional information regarding underwater noise produced by the breaking waves. For brevity, related results are not discussed herein. Relevant studies have presented results regarding this phenomenon. For example, Leighton et al. [5] proposed a technique for determining bubble size and number from underwater noise, and Tien et al. [37] and Tien [38] have subsequently adopted this technique to determine bubble size and number from underwater noise produced by breaking waves near a submerged breakwater, revealing the bubble size distribution in the bubbly flow behind the breakwater. Figure 5 illustrates the experimental setup for the measurement of the air volume fraction in the bubbly flow behind a submerged breakwater by using the proposed fiber-optic sensing system. The sensing system comprises an array of eight fiber-optic probes, a fiber-optic control box, an analog-to-digital (A/D) card, and a personal computer. Figure 6 displays the instruments in the fiber-optic control box.

The fiber-optic control box contains three main units (Figure 6): an emission unit, a sensing unit, and a receiving unit. In the experiments, light with a wavelength of 1550 nm and a power of 1 mW was generated by the laser diode and directed through the isolator and a 1×8 coupler. The isolator protected the light source from backscattered light. The 1×8 coupler split the light beam into eight components, which were then directed through the eight optic fibers into the sensing unit, comprising eight 2×2 couplers, and each of these couplers is connected to the fiber-optic probe. The air or water phase of the medium at the probe tip could be identified because of the differences in their refractive indices. The reflected optical signals were directed through the 2×2 coupler and then to the receiving unit. The photo diodes in the receiving unit converted the optical signals into electronic signals, thus producing the device output. Next, the A/D card was used to convert the analog signals into digital signals (Figure 5), which were then stored on the personal computer.

Table 2 lists the experimental cases investigated in this study. The values for the still water depth, h, incident wave period, T, wave height, H, and breakwater length, b, in Case 1 were the reference values. Cases 2 and 3 were used to examine the effects of wave height on air volume fraction, and Cases 4 and 5 were used to investigate the effects of wave period on air volume fraction. Cases 6 and 7 were used to determine the effects of still water depth on air volume fraction, and Case 8 was used to examine the effects of breakwater length on air volume fraction. The height of the submerged breakwater was 15 cm in all cases. The wavelength, L, listed in Table 2, was determined from the dispersion relationship for small-amplitude waves, as presented in the following equation:

(12) $$\omega^{2}=gk\tanh\;(kh)$$

where ω is the angular frequency (equal to 2π/T) and k is the wavenumber (equal to 2π/L). The wavelength data may provide additional information for understanding the characteristics of the complex bubbly flow behind the submerged breakwater.

In the calibration experiments, to determine the relationship between the air volume fraction, $\beta_{m}$, and the time fraction ratio, α, the probe was always immersed in the water such that the α value was obtained by adding the gas residence time, $T_{Gi}$, of each detected bubble. However, for air volume fraction measurements in the bubbly flow induced by waves propagating over the submerged breakwater, some probes near the free surface might have been exposed to air during the wave trough phase. The measured $T_{Gi}$ value during this time span was not produced by the air bubbles and had to be filtered out. The experimental results revealed that the maximum gas residence time of the air bubbles was approximately 90 ms, which was considerably shorter than the time for which the probe was exposed to air during the phase of wave trough. Since the studied wave period ranged from 0.6 to 1.0 s, the time for which the probe was exposed to free air ranged from 300 to 500 ms. Accordingly, only $T_{Gi}$ values of <90 ms were considered for the air bubbles.

The sampling time in the experiment was 10 s. Since the wave period investigated in this study ranged from 0.6 to 1.0 s, this sampling time was sufficiently long for capturing the main characteristics of the bubbly flow. To perform additional forward measurements, the fiber-optic probe array was moved horizontally at increments of 1 cm in the wave propagation direction. The measurement region stretched 2h in the horizontal direction, where h denotes the still water depth in the wave flume.

## 5. Results and Discussion

### 5.1. Preliminary Experimental Tests

**Figure 7:**
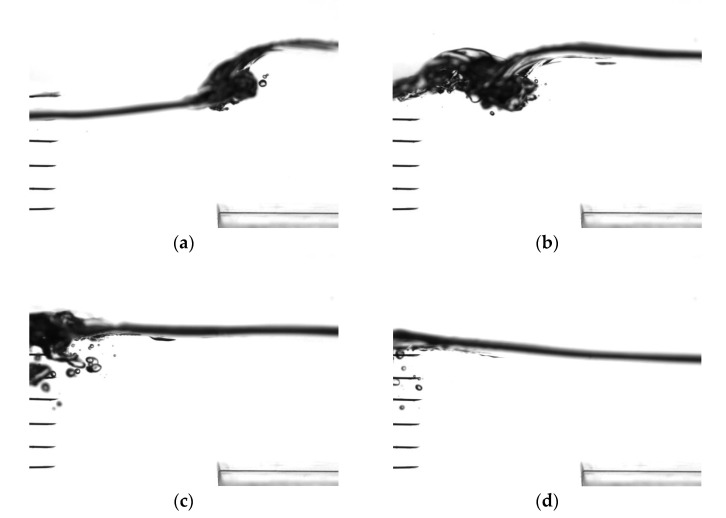
Side view of the motion of the bubbly flow induced by waves propagating over the submerged breakwater in Case 1. The fiber-optic probe array was 10 cm away from the trailing edge of the breakwater. The block in the bottom right corner of each figure panel represents the left corner of the submerged breakwater, which was composed of Plexiglas. (**a**) t/T = 1/10, (**b**) t/T = 2/10, (**c**) t/T = 3/10, and (**d**) t/T = 4/10.

Preliminary tests were conducted to ensure that wave breaking occurred mainly at the lee side of the submerged breakwater and that the probe array was immersed in the bubbly flow. In these tests, a high-speed camera (PIKE F-032B) with a speed of 208 frames per second and a resolution of 640 × 480 pixels was used to capture motion pictures of the bubbly flow behind the breakwater. Figure 7 depicts a side view of the motion of bubbly flow within one wave period that occurred at the lee side of breakwater as waves propagated over the submerged breakwater in Case 1. The block in the bottom right corner of each panel in Figure 7 represents the left corner of the submerged breakwater. As shown in Figure 7, the probe array was 10 cm away from the trailing edge of the breakwater. For brevity, only pictures clearly depicting the bubbly flow are presented. Notably, only six probes were visible because the two lowest probes were located outside the field of vision. Based on this information, the probe array was placed at an appropriate location for measuring air volume fractions. The breaker depicted in Figure 7 can be categorized as a plunging breaker; in such a breaker, the entire wave front steepens, curls, and collapses, and air is entrained into the water to form a bubbly flow. The preliminary tests revealed that wave breaking occurred behind the breakwater in all cases except Case 8, in which the wave began to break on the breakwater because the breakwater was long.

### 5.2. Distribution of Void Fraction in the Bubbly Flows

**Figure 8:**
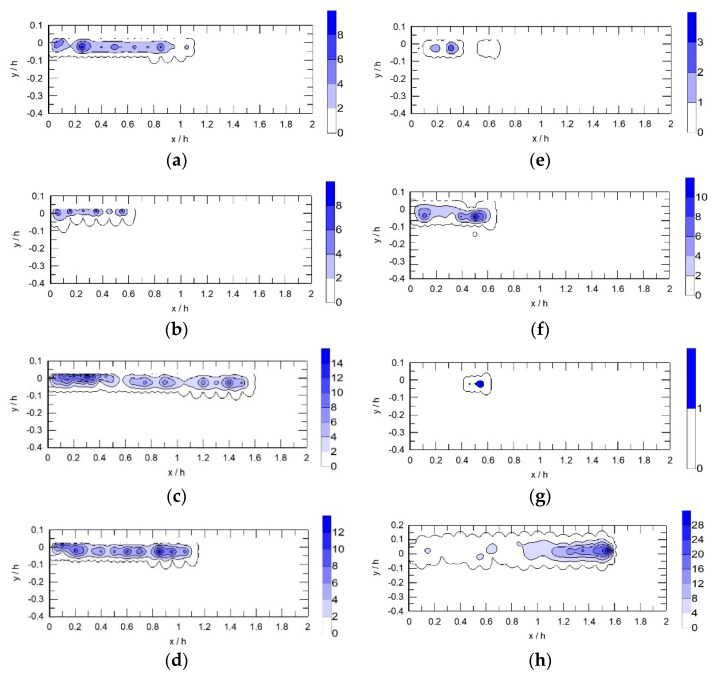
Spatial distributions of the air volume fraction in the bubbly flow at the lee side of the breakwater as waves propagated over the submerged breakwater in Cases 1–8. Here, x=0 represents the trailing edge of the breakwater and y=0 represents the still water level. The waves propagated from left to right. The color bar denotes the air volume fraction. (**a**) Case 1, (**b**) Case 2, (**c**) Case 3, (**d**) Case 4, (**e**) Case 5, (**f**) Case 6, (**g**) Case 7, and (**h**) Case 8.

The time fraction ratios obtained from the experiments were converted into local void fractions by using Equation (10). Figure 8 presents the distributions of the local air volume fraction in the bubbly flows on the rear side of the breakwater in Cases 1–8, where x=0 represents the trailing edge of the breakwater and y=0 represents the still water level. The waves propagated from left to right. When the wave height, H, decreased from 3 cm (Case 1) to 2 cm (Case 2), the air volume fraction and the size of bubbly flow region decreased. When the wave height increased to 4 cm (Case 3), both the air volume fraction and bubbly flow region were considerably higher and larger, respectively, than those in Cases 1 and 2.

When the incident wave period, T, decreased from 1 s in Case 1 to 0.8 s in Case 4 (Table 2), the incident wavelength, L, decreased from 1.212 to 0.888 m. The air volume fraction slightly increased, and the bubbly flow region was approximately the same (Figure 8d). However, when the wave period was further shortened to 0.6 s with an incident wavelength of 0.550 m (Case 5, Figure 8e), the corresponding bubbly flow region and air volume fraction were considerably smaller than those in Cases 1 and 4. The possible reason for this considerable change in the characteristics of the bubbly flow in Case 5 is provided as follows. As depicted in Figure 7, the breaker in Case 1 was a plunging breaker. In coastal engineering, the Iribarren number, also known as the surf similarity parameter, is often used to predict the type of breakers on beaches. According to Battjes [39], for periodic waves propagating on a plane beach, the Iribarren number can be expressed as follows:

(13) $$\xi_{o}=\frac{\tan\gamma_{s}}{\sqrt{H_{o}/L_{o}}}$$

where $\gamma_{s}$ denotes the angle of the seaward slope and $H_{o}$ and $L_{o}$ denote the wave height and length in deep water, respectively. A plunging breaker is formed when $0.5<\xi_{o}<3.3$, and a spilling breaker is formed when $\xi_{o}<0.5$. No unanimous surf similarity parameter is available for breaking waves on a submerged breakwater; however, Equation (13) indicates that a large decrease in wavelength results in a substantial decrease in $\xi_{o}$. When the $\xi_{o}$ value is relatively low, a breaker changes from a plunging breaker to a spilling breaker. In a spilling breaker, the upper part of the crest becomes over-steepened and spills down the front side of the advancing waves. The degree of air entrainment in a spilling breaker is usually considerably smaller than that in a plunging breaker, thus explaining why a considerable decrease in wavelength led to markedly different bubbly flow characteristics (Figure 8e).

As illustrated in Figure 8a,f, a decrease in water depth was associated with a slight increase in the local air volume fraction; nevertheless, the bubbly flow region was located closer to the breakwater. A possible reason for the latter phenomenon is that as the depth of water decreases, the nonlinear effect of waves increases, thus leading to the generation of higher harmonics in addition to the incident first-harmonic wave [40]. Since higher harmonics are associated with shorter wavelengths, therefore, wave breaking occurs closer to the breakwater. In contrast, when the depth of water increases, the nonlinear effect of waves decreases such that nearly no wave breaking occurs. This explains why the bubbly flow region in Case 7 (Figure 8g, h=22 cm) was extremely small.

When the length of breakwater increased from 15 cm in Case 1 to 50 cm in Case 8, the bubbly flow extended to a considerable distance from the breakwater (Figure 8h). The maximum local air volume fraction increased from 8% in Case 1 (Figure 8a) to 28% in Case 8 (Figure 8h), and the main bubbly flow region shifted from x/h=0−0.9 in Case 1 to x/h=1.0−1.6 in Case 8. Huang and Dong [40] studied the deformation of non-breaking water waves propagating over a submerged breakwater and demonstrated that an increase in the breakwater length did not affect the magnitude of the flow separation. Few studies have examined waves breaking near a submerged breakwater. According to our results, the breakwater length considerably affected the air volume fraction distribution. Therefore, the flow separation near the breakwater might not be directly associated with wave breaking. Tien [38] analyzed air bubbles entrained by breaking waves near the same breakwater as that adopted in this study and reported that the number of bubbles in Case 8 was markedly larger than that in Case 1, with considerably smaller bubbles. Small bubbles can be easily transported, which may explain why the bubble flow region in Case 8 was distant from the breakwater.

## 6. Conclusions

This study developed a fiber-optic sensing system with an eight-probe array for determining the spatial distribution of air volume fractions in bubbly flows. First, we derived a calibration curve between the time fraction ratio, α, and the air volume fraction, $\beta_{m}$, in a bubbly flow. Subsequently, we used the proposed fiber-optic sensing system to determine the spatial distribution of the air volume fraction in a bubbly flow induced by breaking waves near a submerged breakwater. The effects of different variables, including incident wave height and period, still water depth, and breakwater length, on the distribution of the air volume fraction of the flow were investigated. Based on our results, we drew the following conclusions related to air volume fractions in bubbly flows induced by breaking waves near a submerged breakwater:

1. The size of the bubbly flow region and the air volume fraction of the flow increased with the incident wave height.
2. When the incident wave period decreased from 1 to 0.8 s the air volume fraction increased. However, when the incident wave period was further reduced to 0.6 s, the bubbly flow region and air volume fraction considerably decreased. This phenomenon can be attributed to the change in the wave breaker type from a plunging breaker to a spilling breaker.
3. As the water depth decreased, the bubbly flow region became closer to the breakwater. This phenomenon can be attributed to the fact that as the depth of water decreases, the nonlinear effect of waves increases, thus resulting in the generation of higher harmonics in addition to the first-harmonic wave. Since higher harmonics are associated with shorter wavelengths, thus, wave breaking occurs closer to the breakwater.
4. When the length of the breakwater increased from 15 to 50 cm, the bubbly flow extended far away from the breakwater, and the maximum air volume fraction increased from 8% to 28%.

## Figures and Tables

**Table 1:** Effect of the sampling time on the curve between the time fraction ratio, α, and the local air volume (void) fraction, $\beta_{m}$, when α<17%.

Sample	$T_{s}$ (s)	Linear Regression	Correlation Coefficient
1	10	$\beta_{m}$=1.17⋅α + 0.0064	0.95
2	5	$\beta_{m}$=1.12⋅α + 0.0103	0.92
3	3	$\beta_{m}$=1.06⋅α + 0.0137	0.89
4	1	$\beta_{m}$=0.97⋅α + 0.0222	0.72

**Table 2:** Experimental cases.

Case	h (cm)	T (sec)	H (cm)	b (cm)	L (cm)
1	20	1.0	3	15	1.212
2	20	1.0	2	15	1.212
3	20	1.0	4	15	1.212
4	20	0.8	3	15	0.888
5	20	0.6	3	15	0.550
6	18	1.0	3	15	1.168
7	22	1.0	3	15	1.252
8	20	1.0	3	50	1.212

h: Still water depth; T: wave period; H: incident wave height; b: breakwater length; L: wavelength, which was determined from the dispersion relation for small-amplitude waves. Height of the submerged breakwater is 15 cm.

## Data Availability

Not applicable.
